# Microbial Functional Potential and Community Composition in Permafrost-Affected Soils of the NW Canadian Arctic

**DOI:** 10.1371/journal.pone.0084761

**Published:** 2014-01-08

**Authors:** Béatrice A. Frank-Fahle, Étienne Yergeau, Charles W. Greer, Hugues Lantuit, Dirk Wagner

**Affiliations:** 1 Alfred Wegener Institute, Helmholtz Centre for Polar and Marine Research, Research Unit Potsdam, Potsdam, Germany; 2 National Research Council, Montréal, Quebec, Canada; 3 University of Potsdam, Potsdam, Germany; 4 GFZ German Center for Geosciences, Section 4.5 Geomicrobiology, Potsdam, Germany; J. Craig Venter Institute, United States of America

## Abstract

Permafrost-affected soils are among the most obvious ecosystems in which current microbial controls on organic matter decomposition are changing as a result of global warming. Warmer conditions in polygonal tundra will lead to a deepening of the seasonal active layer, provoking changes in microbial processes and possibly resulting in exacerbated carbon degradation under increasing anoxic conditions. To identify current microbial assemblages in carbon rich, water saturated permafrost environments, four polygonal tundra sites were investigated on Herschel Island and the Yukon Coast, Western Canadian Arctic. Ion Torrent sequencing of bacterial and archaeal 16S rRNA amplicons revealed the presence of all major microbial soil groups and indicated a local, vertical heterogeneity of the polygonal tundra soil community with increasing depth. Microbial diversity was found to be highest in the surface layers, decreasing towards the permafrost table. Quantitative PCR analysis of functional genes involved in carbon and nitrogen-cycling revealed a high functional potential in the surface layers, decreasing with increasing active layer depth. We observed that soil properties driving microbial diversity and functional potential varied in each study site. These results highlight the small-scale heterogeneity of geomorphologically comparable sites, greatly restricting generalizations about the fate of permafrost-affected environments in a warming Arctic.

## Introduction

Currently there is significant scientific attention being dedicated to the effect of climate warming on permafrost environments, given that they play a crucial role within the global carbon cycle. Current controls of decomposition in such ecosystems are already changing as a result of surface warming, potentially exposing large amounts of previously conserved carbon during the next few decades [1]. Permafrost contains up to 50% of the belowground organic carbon stocks [2], of which a significant part could be lost within the next 100 years due to globally increasing temperatures [3]. Peat wetlands with patterned ground features cover approximately 250,000 m^2^ of permafrost environments [4]. Low-center ice-wedge polygons, which are created by cryogenic processes associated with strong seasonal freeze-thaw cycles [5] typically have a depressed center and an elevated rim, creating a microrelief which affects the hydrology and organic carbon content of the soils. A thickening of the seasonally thawed layer (active layer) of polygons and an ensuing release of previously stored organic matter can stimulate microbial decomposition of this organic carbon, resulting in a positive feedback-loop for global warming [6]. Indeed, peat wetlands are an ideal environment for increased methane production because of the waterlogged, anoxic conditions that prevail in seasonally increasing thawed layers [7]. In wet tundra soils, methanogenesis is the terminal step in the anaerobic decomposition of organic matter and is solely driven by members of *Euryarchaeota*, a subgroup of Archaea which has been identified in numerous permafrost environments [8], [9]. Active methanogenic communities make these environments a significant carbon source through the release of methane to the atmosphere. Methane-oxidizing Bacteria (MOB) present in the aerobic surface layers of the soil and in association with submerged mosses [10], [11] play an important role in the biological oxidation of the methane produced *in-situ*. MOB belong to the *Alpha-* and *Gamma-Proteobacteria* [12] and to the *Verrucomicrobia* [13]. They are able to oxidize up to 90% of the methane emitted in the deeper layers before it reaches the atmosphere [14], [15]. The balance between methane production and oxidation in Arctic environments is sensitive and non-linear as methanogens and methanotrophs have been observed to responds differently to temperature variations in Siberia [16], [17], [18] and Svalbard [19]. Furthermore, accurate predictions of the long-term rates of carbon and nitrogen cycling in Arctic soils, which in turn may determine total ecosystem carbon storage [20], plant productivity [21] and species composition [22], require a much greater understanding of microbial acclimation responses [23]. Yet despite their high relevance in the global climate equation [24], Arctic wetland ecosystems in the Canadian Western Arctic remain poorly characterized in terms of microbial diversity, abundance and the effect of global warming on microbial driven biogeochemical cycles that can result in the net release of greenhouse gases to the atmosphere.

Warmer temperatures in Northern latitudes and the ensuing deepening of the active layer of permafrost can create altered conditions in polygonal tundra, likely causing to major shifts in microbial community composition and their functional potential. This could lead to a strengthening of Arctic wetlands as a carbon source and further reinforce their positive feedback to climate warming.

Therefore, we investigated four polygonal tundra sites on Herschel Island and the Yukon Coast, Western Canadian Arctic. The objectives of our study were to: 1) describe and contrast the bacterial and archaeal diversity in a depth-resolved manner in the four geographically distant polygons; 2) define the essential soil properties shaping this microbial diversity and 3) thereby complement the Circum-Arctic knowledge on permafrost ecosystems in a region insufficiently studied until now. To attain these objectives, we compared and contrasted the microbial communities present in the active layer of four polygonal tundra sites using Ion Torrent sequencing and quantitative PCR. To understand abiotic factors driving this distribution, we described the main soil properties of the sampled profiles. Finally, we used multivariate statistics to elucidate the main biotic and abiotic factors driving the microbial community diversity and structure observed. The results presented give new insights into the distribution and function of microorganisms in a climate-sensitive environment.

## Materials and Methods

### Site description and sample collection

For sampling, the following permissions/permits were obtained: a Parks Canada Research Permit from Ivvavik National Park, Parks Canada; an Exemption from the Environmental Impact Screening Committee, Joint Secretariat of the Inuvialuit Settlement Region; a Research License from Herschel Island Qikiqtaruk Territorial Park, Yukon Parks and a Scientists and Explorers Licence from the Department of Tourism and Culture, Yukon Government.

**Figure 1:**
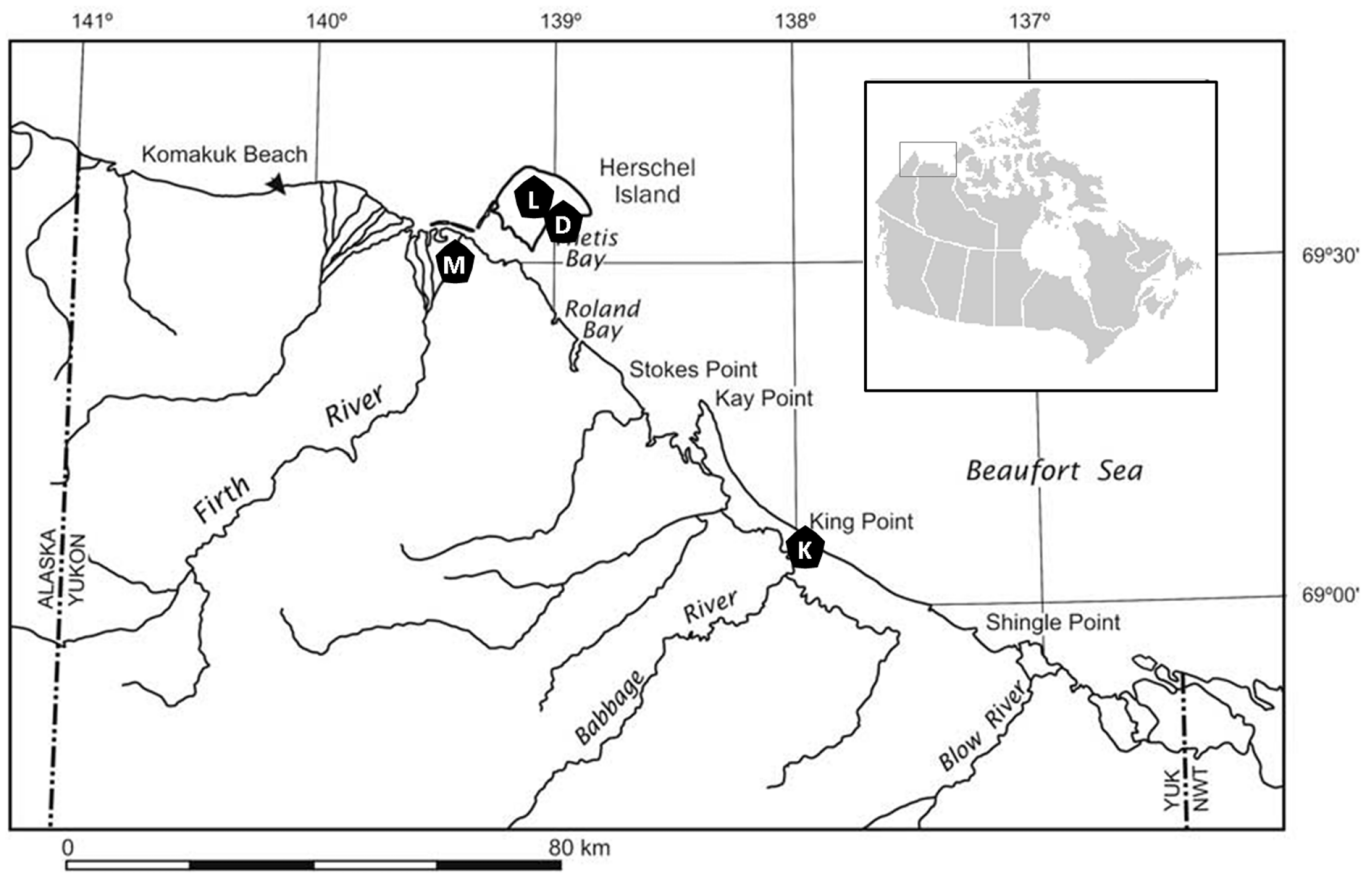
Geographical location of the polygons investigated on Herschel Island and along the Yukon Coast. Map modified from Burn and Zhang, 2009. DP: Drained Lake Polygon, LP: Lake Polygon, MP: Mainland Polygon and KP: King Point Polygon.

Active layer samples were collected from four low-center polygons in the Western Canadian Arctic, on Herschel Island and the Yukon Coast: Drained Lake Polygon (DP, N 69°34′43”, W 138°57′25”), Lake Polygon (LP, N 69°36′00.6”, W 139°03′56.8”), King Point Polygon (KP, N 69°05′26”, W 137°56′45”) and Mainland Polygon (MP, N 69°28′23.7”, W 139°11′06.3”) (Figure 1). The soil at all four sites were characterized as *Hemic Glacistel* classified according to the U.S. Soil Taxonomy [25] with poor drainage and a loamy soil texture. The vegetation period on Herschel Island and the Yukon Coast spans yearly from mid-June to end of September. Average air temperatures vary annually between −26.3°C in February to 8.7°C in July with temperatures at the surface of the active layer ranging from −35°C in the winter to 25°C in the summer [26].

The sampling sites were characterized by an active layer (the layer of ground that is subject to annual thawing and freezing) consisting of a large peat horizon, with a depth varying between 25 cm and 36 cm as measured using a permafrost probe. Replicated sampling was conducted as follows: a large pit was dug in the center of each polygon down to the permafrost table, exposing a clean vertical profile of over a meter in width. At every 5 cm depth, soil samples were taken with a sharp sterile knife to cover small-scale horizontal heterogeneity. Samples obtained from each layer were then homogenized into sterile 125 ml Nalgene® screw-cap containers (Thermo Fischer Scientific Inc., Waltham, MA). The knife was wiped and sterilized with ethanol between different samples. Soil samples were frozen immediately after sampling and stored at −20°C upon arrival in the laboratory. All subsequent subsampling was performed under sterile and anaerobic conditions in an atmosphere-controlled glove box.

### Soil properties

Gravimetric water content of soils was determined by weighing subsamples before and after freeze-drying until no moisture could be observed and the weight stayed constant.

Conductivity and pH were measured using a CyberScan PC 510 Bench Meter (Eutech Instruments Pte Ltd, Singapore) following the slurry technique which consists in mixing 1∶2.5 mass ratio of samples and de-ionized water [27].

The percentage of total organic carbon (TOC) of dried, homogenized soils was measured in duplicate using a TOC analyzer (Elementar Vario max C, Germany) after HCl (10%) acid digestion to remove carbonates.

### DNA extraction and PCR amplification

Total genomic DNA was extracted in duplicate from 0.6 g of soil using the PowerSoil™ DNA Isolation Kit (Mo Bio Laboratories, Carlsbad, CA). Duplicates were then pooled for downstream analyses. The obtained genomic DNA extracts were quantified using the PicoGreen dsDNA quantitation assay (Invitrogen, Carlsbad, CA). DNA was then stored at −20°C for further use. 16S rRNA gene amplification was carried out using multiplex identifiers as described by Bell *et al.* [28] for Bacteria and Yergeau *et al.* [29] for Archaea. Reactions were carried out in 25 µl volumes containing 2 µl of template DNA, 0.5 µM each primer and 12.5 µl of KAPA2G Robust HotStart ReadyMix (KAPA Biosystems, Woburn, MA). Cycling conditions involved an initial 5 min denaturing step at 95°C, followed by 35 cycles of 30 s at 95°C, 30 s at 57°C, and 45 s at 72°C, and a final elongation step of 10 min at 72°C. PCR products were purified on 2.5% w v^−1^ agarose gels using the QIAquick Gel Extraction Kit (Qiagen, Valencia, CA) and quantified using the PicoGreen dsDNA quantitation assay. All 20 amplification products from the different samples were pooled in an equimolar ratio (one pool for Bacteria and one pool for Archaea) and sequenced together. A total of 3.50×10^7^ molecules were used in an emulsion PCR using the Ion OneTouch^™^ 200 Template Kit (Life Technologies, Carlsbad, CA) and the OneTouch^™^ instruments (Life Technologies) according to the manufacturer's protocol. Sequencing of the pooled library was done using the Personal Genome Machine^™^ (PGM) system and a 314 chip with the Ion Sequencing 200 kit according to manufacturer's protocol.

### Quantitative PCR amplification

qPCR was performed in triplicate 20 µl volumes using the iQ SYBR green Supermix (Bio-Rad Laboratories, Hercules, CA, USA) on a Rotor-Gene 3000 apparatus (Corbett Life Science, Sydney, NSW, Australia) as previously described [30]. The primers and temperature conditions are shown in Table 1. Inhibition was checked by diluting the samples until three consecutive dilutions yielded the same results. Standards were made from 10-fold dilutions of linearized plasmids containing the gene fragment of interest that was cloned from amplified soil DNA.

**Table 1:** Primers and temperature profiles used for quantitative PCR analysis.

Functional gene	Primer pair	Temperature (°C) (ann./read)	Reference
*mcrA*	MlF / MlR	55/83	[67]
*pmoA*	A189F / mb661R	55/82	[68]
archaeal *amoA*	amoA19F / crenamoA616r48x	50/72	[69]
bacterial *amoA*	amoA-1F / amoA2R-TC	52/81	[70]
*nifH*	PolF / PolR	55/83	[71]

### Bioinformatic analyses

16S rRNA gene amplicons were pre-treated using the Ribosomal Database Project (RDP) Pyrosequencing pipeline [31]. For Bacteria, sequences having an average quality under 20, having Ns, not exactly matching the MID sequence or being shorter than 100 bp were discarded. For Archaea, sequences having an average quality under 17, having Ns, not exactly matching the MID sequence or being shorter than 75 bp were discarded. The remaining 289658 bacterial and 111596 archaeal sequences were submitted to the RDP classifier [32] using a 0.5 bootstrap cut-off as advised for short sequences [33]. The Shannon diversity index was calculated for each depth for all four study sites. For operational taxonomic unit (OTU) calculations, flowgrams from sff files were de-noised and clustered using AmpliconNoise [34]. Before performing AmpliconNoise calculations, datasets were normalized by randomly selecting 15,000 sequences for Bacteria and 2000 sequences for Archaea. A 97% similarity cutoff was used for OTU calculation, the most stringent OTU definition allowing for intragenomic 16S rRNA variation and PCR/sequencing errors [35].

### Statistical analyses

Weighted-normalized UniFrac distances between each sample pair were calculated using the FastUniFrac website [36] based on the GreenGene core dataset. Most statistical analyses were performed in R (v2.13.2, The R foundation for statistical computing, Vienna, Austria). Spearman rank-order correlations (rs) were carried out using the “cor.test” function. Principal coordinate analyses (PCoA) were carried out using the “cmdcsale” function. Mantel tests based on Spearman correlations were performed using the “mantel” function while permANOVA was performed using the “adonis” function of the “vegan” package. Venny (http://bioinfogp.cnb.csic.es/tools/venny/index.html) was used to draw Venn diagrams from genus presence-absence data.

### Data deposition

All sequence data have been submitted to the NCBI sequence read archive (SRA) database under project SRP026122 (BioProject PRJNA208722).

## Results

### Characteristics of the soil

The permafrost table was measured at 36 cm depth for DP, 25 cm for KP, 26 cm for LP and 36 cm for MP in the period from July 27^th^ to August 2^nd^, 2010. All polygon sites were visibly water saturated, with gravimetric water contents ranging from 65 to 86%, with the exception of the two lower depths of KP (41–42%). Within each polygon, water content did not significantly correlate with depth. The pH at polygons DP and MP was pH 5.4 on average, slightly more basic at LP (pH 5.7 to 6.8) and more acidic at KP (average pH 4.8), and was only significantly correlated with depth in the MP polygons (r_s_ = −0.874; *P* = 0.0045). Total organic carbon content (TOC) was high at all sites, ranging from 20 to 40%, with the exception again of the bottom layers of KP where TOC was lower (10–11%). TOC was only significantly correlated with depth in the KP polygons (r_s_ = −1.00; *P* = 0.017). Soil C:N ratio was significantly and positively correlated with depth in the DP (r_s_ = 0.943; *P* = 0.017) and MP (r_s_ = 0.881; P = 0.0072) polygons while it showed significant negative correlation with depth in the KP polygons (r_s_ = −1.00; *P* = 0.017). An overview of the soil properties of each polygon is given in Table 2.

**Table 2:** Main soils properties of the four polygons investigated.

Name of polygon GPS coordinates	GPS Coordinates	Depth (cm)	Water content (%)	N (%)	C (%)	C:N	TOC	pH	Conductivity TRef. 25°C (µS.cm^−1^)
Drained Lake	N 69°34′43”	5–10	79	1.79	26.65	14.89	24.48	5.3	68.1
Polygon (DP)	W 138°57′25”	10–15	77	1.89	30.63	16.21	28.24	5.46	54.9
		15–20	78	1.59	30.44	19.14	28.29	5.53	59.0
		20–25	76	1.18	24.12	20.44	25.20	5.44	62.8
		25–30	79	1.19	26.43	22.21	23.19	5.23	74.3
		30–35	83	1.25	27.62	22.1	26.04	5.29	85.9
King Point	N 69°05′26”	0–5	82	1.01	40.09	39.69	38.23	4.33	111.7
Polygon (KP)	W 137°56′45”	5–10	84	1.11	38.22	34.43	37.75	4.47	91.8
		10–15	86	1.11	35.91	32.35	34.31	4.96	101.7
		15–20	41	0.54	13.27	24.57	11.30	5.19	28.0
		20–25	42	0.25	6.04	24.16	9.77	5.08	39.2
Lake Herschel	N 69°36′00.6”	5–10	76	1.35	25.08	18.58	22.69	6.82	76.4
Polygon (LP)	W 139°03′56.8”	10–15	76	1.33	24.92	18.74	22.66	5.73	84.7
		15–20	69	1.14	20.62	18.09	16.56	5.83	72.5
		20–25	77	1.76	29.77	16.91	28.09	5.9	91.3
Mainland	N 69°28′23.7”	0–5	79	2.35	33.36	14.2	31.76	5.54	45.7
Polygon (MP)	W 139°11′06.3”	5–10	80	2.49	39.61	15.91	38.25	5.47	40.5
		10–15	80	2.68	42.69	15.91	39.20	5.46	38.6
		15–20	76	1.78	31.95	17.91	29.95	5.41	45.0
		20–25	68	1.45	26.32	18.15	24.08	5.44	53.4
		25–30	65	1.22	23.55	19.30	21.32	5.46	49.3
		30–36	78	2	35.96	17.98	34.57	5.39	57.2

### Quantitative PCR

**Figure 2:**
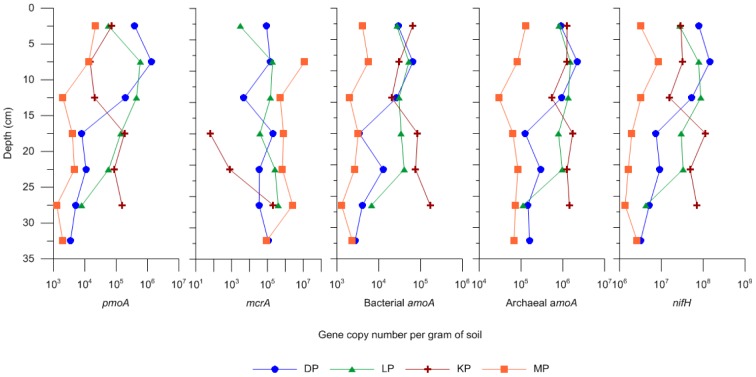
Abundance of selected functional genes in the active layer. Gene copy numbers per gram of soil in the active layer profile of each site investigated as determined by qPCR for *pmoA*, *mcrA*, bacterial *amoA*, archaeal *amoA* and *nifH* genes.

The functional genes investigated were *pmoA* (subunit A of particulate methane monooxygenase), *mcrA* (methyl coenzyme-M reductase), bacterial and archaeal *amoA* (ammonia monooxygenase) and *nifH* (nitrogenase reductase subunit). Overall, gene copy numbers per gram of wet soil were abundant for all of the functional genes investigated and in all four polygons. The only exception is the complete absence of an amplifiable *mcrA* signature in the first 15 cm depth in KP (Figure 2). *nifH* was consistently the most abundant of all of the genes analysed in all four polygons, reaching up to 10^8^ copies per gram of wet soil (Figure 2). *nifH* abundance was negatively correlated with depth and C:N ratio in the DP polygons (r_s_ = −0.952; *P* = 0.0011 and r_s_ = −0.886; *P* = 0.033, respectively) and in the MP polygons (r_s_ = −0.786; *P* = 0.028 and r_s_ = −0.881; *P* = 0.0072, respectively). Furthermore, in the MP polygons, the abundance of *nifH* was significantly and positively correlated with the concentration of N and C (r_s_ = 0.952.786; *P* = 0.0011 and r_s_ = 0.929; *P* = 0.0022, respectively). Archaeal *amoA* was found to have between 10^5^ to 10^6^ copies.g^−1^, while bacterial *amoA* was one or two orders of magnitude lower (10^3^ to 10^4^ copies.g^−1^, Figure 2). In the DP polygons, both archaeal and bacterial *amoA* were negatively correlated with depth (r_s_ = −0.762; *P* = 0.037 and r_s_ = −0.905; *P* = 0.0046, respectively), in the KP polygons the abundance of bacterial *amoA* was negatively correlated with soil water content (r_s_ = −1.00; *P* = 0.017), while in the MP polygons, bacterial *amoA* was negatively correlated with depth and C:N ratio (r_s_ = −0.786; *P* = 0.028 and r_s_ = −0.786; *P* = 0.028, respectively). *mcrA* was more erratically distributed throughout the different depths and varied more between polygons, with copy numbers ranging anywhere from 10^1^ to 10^7^ copies g^−1^. *mcrA* only showed significant correlations with soil properties in the KP polygons, where it was significantly correlated to depth, N, C and C:N ratio (r_s_ = 0.941; *P* = 0.0051, r_s_ = −0.918; *P* = 0.028, r_s_ = −0.894; *P* = 0.041 and r_s_ = −0.894; *P* = 0.041, respectively). *pmoA* only showed significant correlations in the DP polygons, where it was negatively correlated with depth and C:N ratio (r_s_ = −0.952; *P* = 0.0011 and r_s_ = −0.886; *P* = 0.033, respectively).

### Biodiversity and richness index

**Figure 3:**
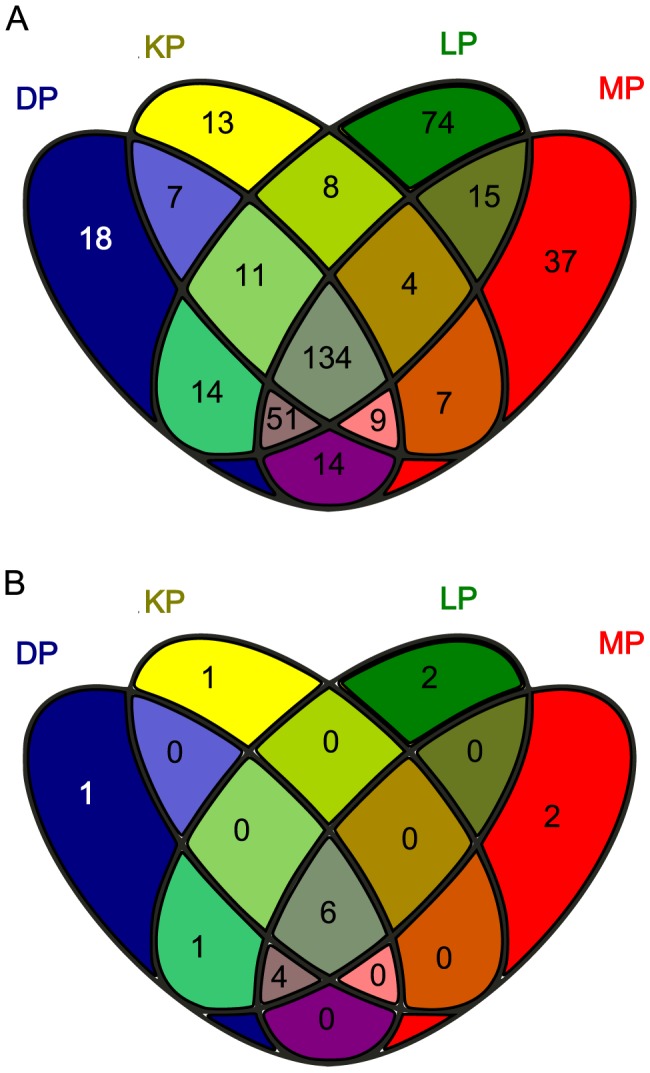
Venn diagrams illustrating the distribution of identified taxa between sampling locations. a) Distribution of classified bacterial genera between polygons. b) Distribution of classified archaeal genera between polygons.

The bacterial and archaeal diversity was high, with an overall Shannon index (H’) of 5.8 and 4.7, respectively. In all polygons, the bacterial diversity was highest in the surface samples, then decreasing with depth (Table 3). Archaeal diversity was highest in the upper layers of the soils, the maximum diversity found between 0–15 cm depth (Table 3). LP showed the highest diversity overall with H’6.2 for Bacteria and 4.9 for Archaea. Based on OTU (0.03) calculations, the highest number of bacterial OTUs was found in DP, while the highest number of archaeal OTUs was found in LP (Table 3). Out of a total of 416 bacterial genera identified in all polygons combined, 134 were shared by all four sites. DP harboured 18 unique genera, KP 13, LP 74 and MP 37 (Figure 3a). However, due to the short length of the archaeal 16S rRNA sequences and the lower number of observed archaeal genera, only 12 genera could be identified, of which 6 were shared between all polygons (Figure 3.b., grey colored field). Interestingly, 4 genera were shared between all polygons except KP.

**Table 3:** OTU (0.03) calculations and Shannon diversity index H’ (in parentheses) of bacterial and archaeal 16S rRNA gene sequences from the active layer of four low-center polygons on Herschel Island and the Yukon Coast.

	DP		LP		KP		MP	
Depth (cm)	Bac.	Arch.	Bac.	Arch.	Bac.	Arch.	Bac.	Arch.
0–5	8951	863	7889	1446	9084	N.a.	9207	1043
	(6.3)	(4.8)	(6.6)	(5.8)	(5.7)		(6.2)	(5.1)
5–10	9086	953	9215	1392	8586	N.a.	799	1023
	(6.2)	(5.0)	(6.4)	(5.7)	(5.7)		(6.1)	(5.1)
10–15	7453	1027	8317	1111	5379	N.a.	7892	1007
	(6.2)	(5.1)	(6.2)	(5.1)	(5.9)		(5.7)	(4.7)
15–20	9155	851	7856	971	6974	1040	7171	700
	(6.0)	(4.6)	(5.8)	(4.7)	(5.1)	(5.2)	(5.6)	(4.6)
20–25	8762	864	8829	767	5349	837	5923	933
	(5.8)	(4.3)	(6.3)	(4.3)	(5.1)	(4.7)	(5.7)	(4.5)
25–30	8258	937	N.d.	N.d.	N.d.	N.d.	7326	825
	(5.6)	(4.4)					(5.2)	(4.5)
30–35	8084	900	N.d.	N.d.	N.d.	N.d.	7802	821
	(5.5)	(4.2)					(5.5)	(4.6)
**Average**	**8512**	**887**	**7736**	**1064**	**7140**	**934**	**7706**	**869**
	**(5.9)**	**(4.5)**	**(6.2)**	**(4.9)**	**(5.4)**	**(4.8)**	**(5.7)**	**(4.7)**

N.d. refers to no sample being taken as the permafrost table has been reached. N.a. refers to no amplifiable sequence being recovered from those samples.

### Community composition and vertical distribution of diversity

**Figure 4:**
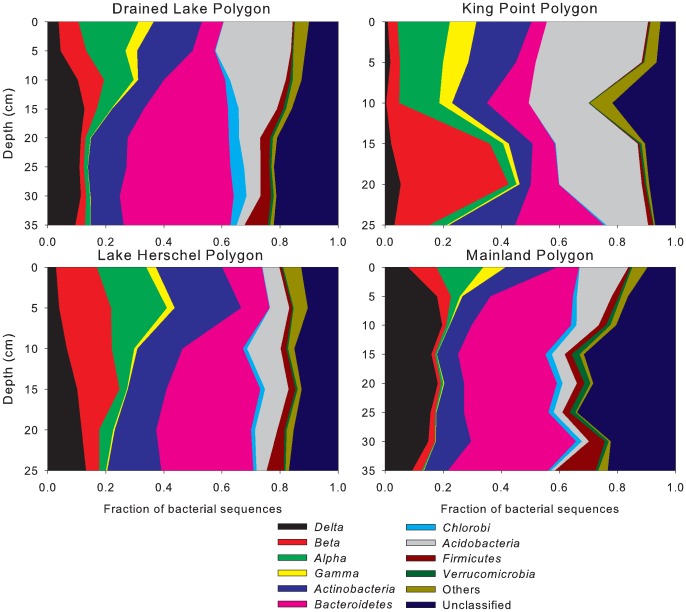
Distribution of bacterial phyla with depth based on 16S rRNA sequences in all four polygons. Alpha, Beta, Delta and Gamma stand for *Alphaproteobacteria*, *Betaproteobacteria*, *Deltaproteobacteria* and *Gammaproteobacteria*.

A bootstrap cutoff of 50% was shown to accurately classify sequences at the genus level for partial sequences of length longer than 50 bp and shorter than 250 bp, and to provide genus level assignments for higher percentage of sequences [33]. Bacterial sequences were classified to the phylum level, also detailing the proteobacterial classes. Archaea were classified to the genus level. The bacterial community composition at all four sites were found to be similar in terms of overall diversity, but the community evolved differently with depth (Figure 4). Sequences affiliated with *Proteobacteria* accounted for 40–50% of sequences in all sites, followed by *Bacteroidetes*-affiliated sequences at 20–40% and *Actinobacteria* at 10–15%.

Communities in all four polygons showed a similar evolution of community composition with depth: *Deltaproteobacteria*, *Bacteroidetes* and *Firmicutes* increasing in abundance and *Alpha-, Beta-, Gammaproteobacteria* and *Acidobacteria* decreasing with depth (Figure 4).

**Figure 5:**
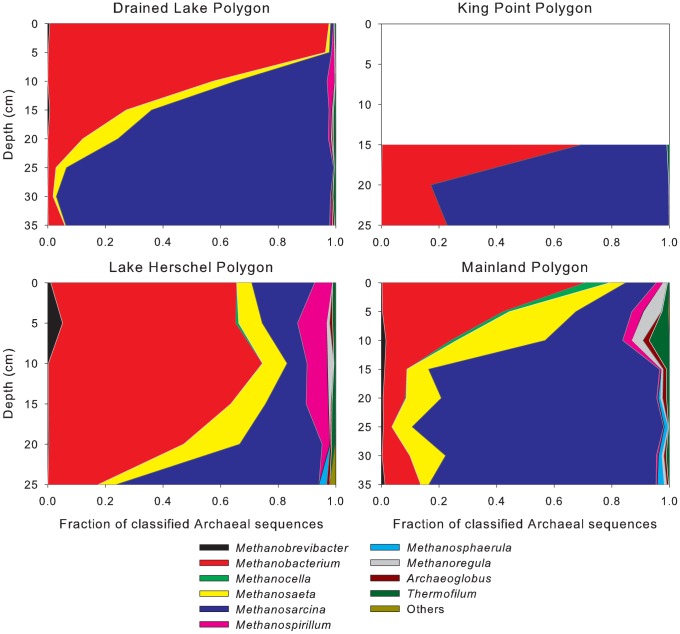
Distribution of archaeal genera with depth based on 16S rRNA sequences in all four polygons. No archaeal 16S rRNA sequence could be amplified between 0 and 15(KP).

The class-level compositions of Archaea were similar in DP and MP (Figure 5). No archaeal sequences could be amplified in KP from the first 15 cm of the active layer. Sequences affiliated with methanogens (*Methanobacterium* and *Methanosarcina*) dominated in all sampling sites, representing up to 90% of all classified sequences. Within the classified sequences, *Methanobacterium* were found to clearly dominate in the surface layers of three out of the four polygons, representing up to 98% of classified sequences in DP. They decreased rapidly with depth, replaced by a majority of *Methanosarcina* and to a lesser extent *Methanosaeta*, representing more than 80% of the community in the deeper layers. The exception was LP, where the abundance of *Methanobacterium* remained relatively constant to a depth of 20 cm after which *Methanosarcina* became more abundant.

**Figure 6:**
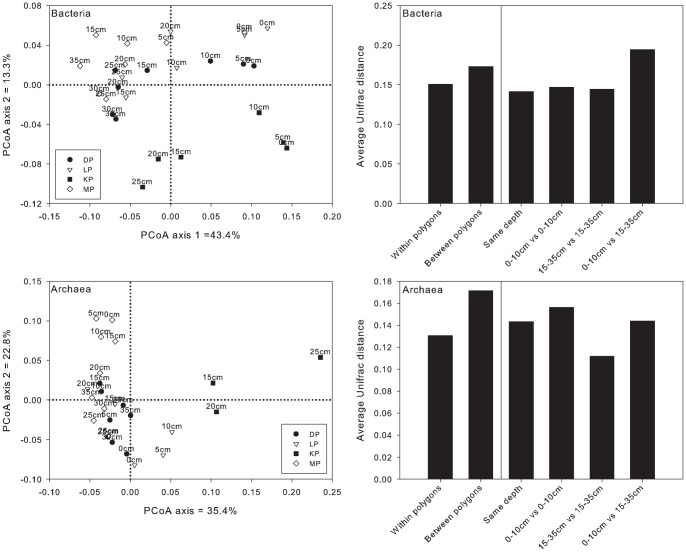
Unifrac analysis of 16S rRNA sequence variation within and between polygons.

Overall, little variation was observed for the bacterial community between the polygons based on UniFrac values, however the variation within each polygon (i.e. with increasing depth) was higher. On the contrary, the archaeal community composition was more influenced by location, each polygon having an own, specific community. For Bacteria, the KP samples separated from all the other samples (Figure 6). Another interesting trend was the separation of the majority of deep samples (15–35 cm deep) versus the majority of the shallow samples (0–10 cm deep). This trend was also visible when looking at the average UniFrac distance between shallow and deep samples, which was relatively high. For Archaea, again the KP samples clustered separately from all other samples. The 15 to 35 cm deep samples tightly clustered together, as made visible by the relatively low UniFrac distance between samples from that depth. For both Archaea and Bacteria, the microbial communities were more similar within a polygon then between polygons (UniFrac distances), indicating some level of local adaptation.

**Figure 7:**
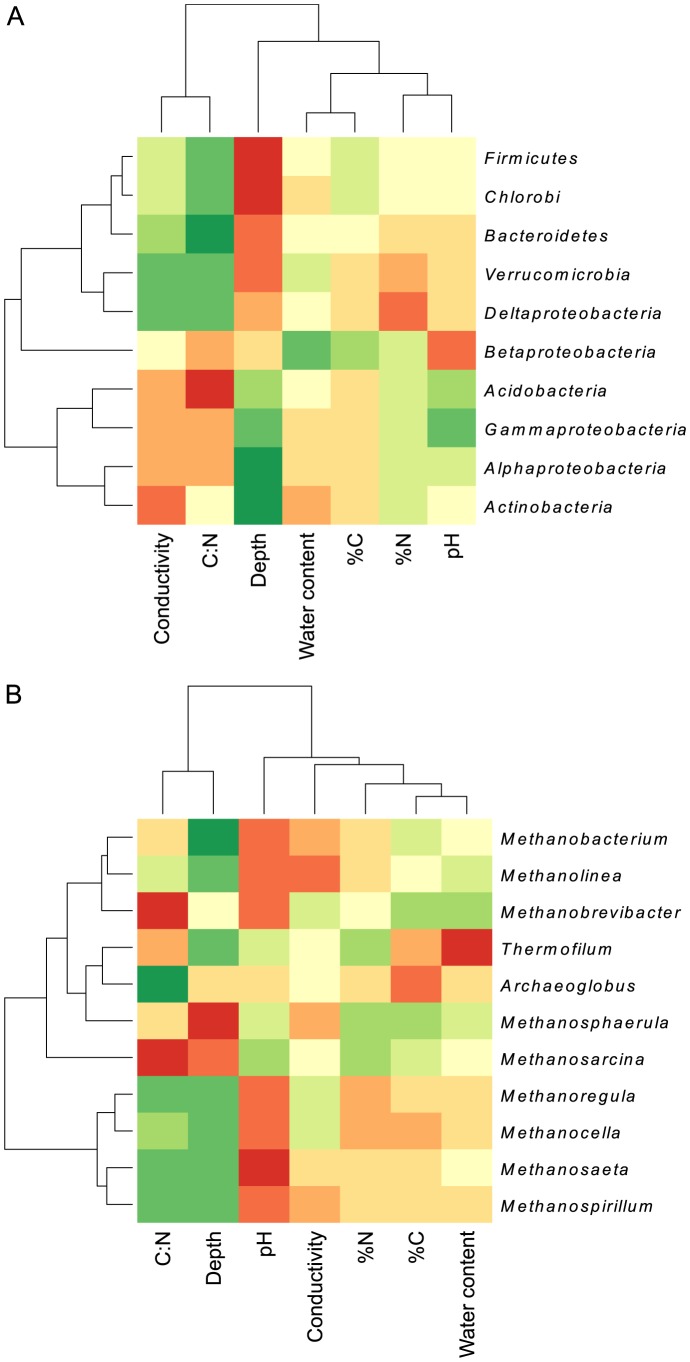
Heat map correlation between the taxonomical distribution of microorganisms and the main properties of the soil. a) Correlations between soil properties and bacterial phyla. b) Correlations between soil properties and archaeal genera. A red colour indicates a positive correlation, while a green color indicates a negative correlation. The darker the colour, the stronger the correlation.

To investigate potential relationships between microbial community structure and the underlying soil geochemistry, the sequence data were correlated to the soil properties data and illustrated by heat maps (Figure 7). Variables most consistently explanatory of bacterial sequence diversity patterns were depth, conductivity and C:N ratio. Archaeal community composition showed a stronger positive correlation to increasing pH, with depth and C:N also having a strong influence on community structure.

## Discussion

Increasing temperatures in the Northern latitudes and the ensuing deepening of the active layer of permafrost will likely lead to an increase in the anoxic zone and thereby shift microbial community composition and functional potential. Several studies have addressed aspects of this issue in Siberia (for example [6], [8], [37]), Svalbard [19], [38] and the Canadian High Arctic [9]; [39] but the communities of the Canadian Western Arctic remain underexplored [40]. This study represents the first local-scale comparative microbiological analysis of polygonal tundra in the North-American Arctic. We found that depth strongly influenced microbial community in terms of structure and function, this effect being related to an increase in C:N ratio (and thus nutrient limitation) in deeper layers. This depth-effect has previously been described by Wagner *et al.* [41] who observed a net decrease of bioavailable organic carbon and an increasing humification index with increasing soil depth in Siberian polygonal tundra. The authors measured the highest rates of methanogenesis at depths with the highest amounts of bioavailable carbon rather than total organic carbon. Their findings concur with ours, indicating that although organic carbon is highly accumulated in permafrost soils, there is a decrease in bioavailable carbon with increasing depth, creating substrate limitation close to the permafrost table. Looking at the microbial diversity in the polygons, a large number of microbial genera were shared between all sites despite the sites being up to 70 km apart as illustrated by the Venn diagrams (Figure 3). However, these diagrams also show that an important number of genera identified were unique to each polygon, suggesting that although the bulk of the community is similar across our study sites, each polygon contains a number of unique microorganisms whose presence is supported by specific local environmental characteristics. The shift in community composition with active layer depth at all sites suggests a certain level of local adaptation. Within each polygon, we saw highly parallel trends, with increasing *Bacteroidetes* and decreasing *Alpha-* and *Betaproteobacteria* in samples closer to the permafrost table, with the exception of increasing *Betaproteobacteria* with depth in KP. Although they represent 20 to 40% of sequences obtained in all four polygons, the considerable variation within *Proteobacteria* with depth indicates consistent forcing mechanisms leading to the adaptation and specialization of the various groups to changes in temperature and soil properties. In deeper layers, the dominance of *Bacteroidetes* was not surprising as these microorganisms are known to include several psychrophilic members [42] which thrive under the stable cold conditions found in the deeper active layer close to the permafrost table. A study of polygonal tundra in the Lena Delta, Siberia also identified *Bacteroidetes* as dominant members of the soil microbial community [37]. Commonly found in permafrost-affected soil ecosystems [43], [44], *Actinobacteria* also made-up an important part of the bacterial community at our sites, their successful colonization of the entire active layer likely being supported by their ability to metabolize a wide range of substrates as sole carbon source and their adaptation to low temperatures [45], [46]. Overall, these results are consistent with other studies reporting that environmental conditions found in the active layer of permafrost-affected soils permit a diverse community of Bacteria, capable of utilizing a wide spectrum of substrates under varying redox conditions [44]. The redox potential in the active layer of permafrost environments has consistently been shown to decrease with depth, effectively shaping the structure and activity of the indigenous microbial community [37], [44], [47], [48].

In the active layer, the final step of organic carbon decomposition under anaerobic conditions is catalyzed by methanogenic Archaea. At our study sites the methanogens were distributed throughout the soil profile, this dominance being in line with the archaeal diversity described in peat wetlands in Siberia and Svalbard [8]; [49]. The archaeal community composition was similar at phylum level between DP, LP and MP but significantly different in KP. In all four polygons, it appears that the large shifts in archaeal community composition with depth observed had no apparent effect on the functional potential for methane emission as measured by quantitative PCR. This suggests a certain amount of functional redundancy, defined as species performing similar roles in communities and ecosystems and therefore being substitutable with little impact on ecosystem processes [50]. The strong difference in archaeal community structure observed at KP can be attributed to the lower pH, as suggested by the fact that only a few acidophilic methanogens have been cultured so far [51]; [52] and the positive correlation between increasing pH and archaeal community composition found in this study. The higher conductivity found in the top layers of KP due to increased sea spray because of the proximity of the sampling site to the Beaufort Sea could also have an inhibitory effect on Archaea in the surface of KP.

We found that both hydrogenotrophic and acetoclastic methanogenesis can occur in the active layer. The dominance of *Methanobacteria* in the top layers can be attributed to their use of hydrogen as a substrate, as hydrogenotrophy is especially dominant at higher temperatures [53]. The methanogenic community shifted to a *Methanosarcina* dominated community with depth in three of our study sites. *Methanosarcina*-related sequences are readily detected in permafrost-affected soils and sediments [17], [54], one reason for this ubiquity being the potential of *Methanosarcina*-like species to use all three metabolic pathways with a broad range of substrates, e.g. acetate, methanol and hydrogen [55]. This provides *Methanosarcina* with a high flexibility regarding changing substrate and environmental conditions. *Methanosarcina* species also show a high resistance against several abiotic and biotic stress factors, as recently illustrated by the new isolated strain *Methanosarcina soligelidi* [56], [57]. The sharp switch of the methanogenic community from a *Methanobacteria* to a *Methanosarcina* dominated community, i.e. a switch from an overall hydrogenotrophic to an acetoclastic methanogenic community, has been observed through cloning and T-RFLP analysis in the detailed study of a polygon in the same area [40] and in other Arctic tundra environments [8], [49], [58]. Our findings further support the hypothesis that the acetoclastic pathway is favored in cold environments [53] because of low temperatures inhibiting hydrogen-producing Bacteria. Furthermore in cold, anoxic conditions such as those encountered close to the permafrost table, hydrogenotrophic methanogenesis is disadvantaged because of competition for hydrogen and carbon dioxide with acetogenic bacteria (e.g. *Bacteroidetes*) which produce acetate as a precursor for acetoclastic methanogens [59]. The availability of low molecular substances (e.g. acetate) provided by the root system of the vegetation [60], [61] could also influence the composition of the methanogenic community.

The investigation of nitrogen and carbon cycling genes revealed the possible functional evolution of the microbial community with respect to increasing active layer temperatures. Several of the genes quantified decreased with increasing depth, suggesting a lower functional potential in the deeper layers of the polygons. The depth effect we observed was not caused by increasing water saturation in deeper layers as was expected. Rather, we observed that various soil factors were the most likely explanatory factors for microbial functional potential, and that this varied largely depending on the polygon. The decrease observed is proposed to be linked with the decreasing C:N ratio and the concomitant increased substrate limitation with active layer depth as discussed above for bacterial community composition. The abundance of functional genes involved in nitrogen fixation, ammonium oxidation, methane production and methane oxidation was found to be higher by one to two orders of magnitude compared what has been reported from Hess Creek, Alaska [62] and the Canadian High Arctic [9], [39]. The high abundance of nitrogen fixers and ammonia oxidizers in the active layer has already been described by previous studies [9], [63] especially the majority of archaeal compared to bacterial *amoA*, in our case by almost two orders of magnitude. The dynamics of nitrogen processes may be especially important, as most tundra ecosystems are considered to be nitrogen limited and changes in nitrogen supply can affect leaf development, carbon flux and biomass production [64], [65]. Genetic proxies for methane production and oxidation, *mcrA* and *pmoA* respectively, were both found in large amounts at virtually all depths of the active layer at all sites except KP. The presence of aerobic methane oxidizers also in deeper, water saturated layers has important implications for mitigating the release of methane to the atmosphere, their presence likely being supported through root exudates creating microaerobic conditions favorable for methane oxidation [66].

In summary, this study provides an important insight into the microbial functional role of Arctic permafrost-affected soils. We determined that soil water content does not have the expected influence on microbial communities, but that depth related changes in soil parameters have a strong influence in shaping microbial abundance and distribution. The future of carbon turnover in permafrost ecosystems largely depends on the structure and functional potential of the indigenous microbial community within the permafrost and the overlaying active layer. Other studies of permafrost-affected environments have determined that although these ecosystems are apparently similar in many aspects, their microbial community composition and constraints on organic matter turnover can vary greatly, emphasizing the need to conduct more circum-Arctic studies. Our results highlight the very large heterogeneity of polygons, as the microbial community composition and functional potential of each seemingly identical polygon responded to a different set of environmental controls. So, even if they appear to be highly similar, Artic polygon microbial communities and functions are under different environmental controls at each location. This heterogeneity makes the prediction of the fate of the carbon stocked in these ecosystems and generalizations across landscapes very difficult, if not impossible, and calls for more in depth studies like the current one.
